# Antioxidant activity against H_2_O_2_-induced cytotoxicity of the ethanol extract and compounds from *Pyrola decorate* leaves

**DOI:** 10.1080/13880209.2017.1333126

**Published:** 2017-06-01

**Authors:** Xiliang Yang, Qingyun Peng, Qian Liu, Jie Hu, Zhipeng Tang, Lianjie Cui, Zonghao Lin, Bing Xu, Kuojian Lu, Fang Yang, Zhizheng Sheng, Qiong Yuan, Song Liu, Jiuliang Zhang, Xuefeng Zhou

**Affiliations:** a Department of Pharmacy, Medical College, Wuhan University of Science and Technology, Wuhan, China;; b College of Food Science and Technology, Huazhong Agricultural University, Wuhan, China;; c Key Laboratory of Tropical Marine Bio-Resources and Ecology, Guangdong Key Laboratory of Marine Materia Medica, South China Sea Institute of Oceanology, Chinese Academy of Sciences, Guangzhou, China

**Keywords:** Pyrolaceae, terpenoids, oxidative stress, isolation

## Abstract

**Context:** The leaves of *Pyrola decorate* H. Andr (Pyrolaceae), known as Luxiancao, have long been used for treating kidney deficiency, gastric haemorrhage and rheumatic arthritic diseases in traditional Chinese medicine.

**Objective:** The phytochemicals and antioxidant capacities *in vitro* of *P. decorate* leaves were investigated.

**Materials and methods:** Ethanol, petroleum ether, acetidin, *n*-butyl alcohol and aqueous extracts of *Pyrola decorate* leaves were prepared by solvent sequential process, and then isolated and purified to obtain phytochemicals. Cell viability was measured by MTT assay. PC12 cells were pretreated for 24 h with different extractions of *P. decorate* leaves at concentrations of 0.1, 0.5, 1, 5 and 10 mg/mL, then H_2_O_2_ of 0.4 mM was added in all samples for an additional 2 h. The antioxidant capacities of betulin, ursolic acid and monotropein were determined in PC12 cells against H_2_O_2_ induced cytotoxicity *in vitro* as well.

**Results:** Nine compounds (**1**–**9**) were isolated and structurally determined by spectroscopic methods, especially 2D NMR analyses. Ethanol extract treated groups showed inhibitory activity with IC_50_ value of 10.83 mg/mL. Betulin, ursolic acid and monotropein were isolated from *P. decorate*, and demonstrated with IC_50_ values of 6.88, 6.15 and 6.13 μg/mL, respectively.

**Discussion and conclusions:** In conclusion, *Pyrola decorate* is a potential antioxidative natural plant and worth testing for further pharmacological investigation in the treatment of oxidative stress related neurological disease.

## Introduction

*Pyrola* herb, with about 30 species, has the widest distribution occurring in the northern hemisphere in temperate and cold temperate regions around the world, while 27 species are distributed mainly in the west and northeast part of China (Yao et al. 2013). As a Yang-tonic agent, *Pyrola decorate* H. Andr (Pyrolaceae), also known as Luxiancao or Luticao, has been extensively used as a valuable tonifying agent for more than 2000 years in China. It has been included in Shengnong’s herbal classic and came out of the top grade lists. According to traditional Chinese medicine (TCM) theory, tonic herbs have been used for various patterns of body deficiency and anti-aging (Ho et al. 2009). *P. decorate* has been used to nourish ‘kidney’ and strengthen ‘bone and muscle’ for long history. Therefore, it has been used as treatment for kidney deficiency, gastric haemorrhage and rheumatic arthritic diseases in Chinese medicine (Zhang et al. 2013).

As a tonifying agent, *P. decorate* is an important component in many Chinese prescription formulas for aging-associated diseases, such as Alzheimer’s disease (AD), Parkinson’s disease (PD) and other neurodegenerative diseases (Luo et al. 2004). Leaves of *Pyrola* are rich in a variety of active components, such as triterpenoids, flavones polysaccharides, phenolics glycosides, quinines and tannic acid (Ptitsyn et al. 2011; Zhang et al. 2012; Kirillov et al. 2015). However, detailed pharmacological evidence on *Pyrola decorate* need to be further elucidated. Our previous studies have shown the different polar solvent extracts from the leaves of *P. decorate* showed various neuroprotective effects against the Aβ_25–35_ induced apoptosis in PC12 cells, with the petroleum extraction (PE) and acetidin extraction (AE) showing better neuroprotective effects than other extractions. Oxidative stress is an early and sustained event in neurodegenerative disease progression (Uttara et al. 2009), and plays a significant role in many neurological diseases, such as AD, PD and cerebral ischemia (Saeidnia & Abdollahi 2013). The ‘free radical theory of aging’ shows promise in helping to understand the process of aging and in treating age-related diseases. Therefore, the antioxidant effect of *P. decorate* leaves might be one of the underlying mechanisms for neuroprotective effects. It is very important to protect neuronal cells from oxidative injury for the treatment of neurodegenerative disorders.

**Figure 1.**
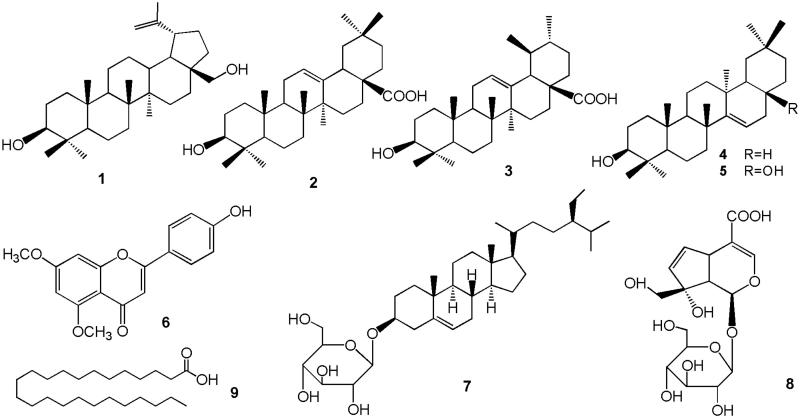
Chemical structures of compounds **1**–**9**.

As our continuous investigation of *P. decorate* active constituents, further purification and isolation study was conducted. Nine compounds were isolated and identified from the extracts of *P. decorate* leaves, including five triterpenoids, an iridoid, a flavone, a sterol and an aliphatic acid (Figure 1). In addition, we investigated *in vitro* antioxidant capacities of *P. decorate* decoction, as well as the isolated phytochemicals, in protecting PC12 cells from hydrogen peroxide induced oxidative stress. Our study provides some basis of mechanisms in prevention and treatment of neurodegenerative diseases including AD and PD by *P. decorate*.

## Materials and methods

### General experimental procedures

^1^H-, ^13^C NMR, COSY, HMQC spectra were recorded on a Bruker AV500 NMR spectrometer (TMS as internal standard, ^1^H 400 MHz, ^13^C 100 MHz). HR-ESI-MS was conducted on an Agilent (Santa Clara, CA) 1100 LC/TOF/MSD system (ESI model), Shimadzu (Kyoto, Japan) LC-10 ATVP HPLC with ODS-AGG12 (YMC, Kyoto, Japan) column, column chromatography and TLC silica gel (Qingdao Marine Chemical Group Co., Qingdao, China). All the solvents used in the isolation and purification studies were analytical grade.

Culture PC12 cell lines were gifted from the College of Animal Science and Technology, Huazhong Agriculture University (Wuhan, China). Foetal bovine serum (FBS) was purchased from Hangzhou Tianhang Bio-Technology Co., Ltd. (Hangzhou, China) and the cell culture medium (DMEM) was purchased from Hyclone (Logan, UT). 3-(4,5-Dimehthylthiazol-2-yl)-2,5-diphenyltetrazolium bromide (MTT) and lipopolysaccharide (LPS) were obtained from Sigma (St. Louis, MO).

### Plant material

The *P. decorate* leaves were collected in September 2013 in Wuhan, China, and were identified by Professor Yongzhong Zhang (Department of Pharmacognosy, Medical College, Wuhan University of Science and Technology, Wuhan, China). A voucher specimen (No. 130901-01) was deposited in Department of Phytochemistry, Medical College, Wuhan University of Science and Technology, Wuhan, China.

### Extraction and isolation

The *P. decorate* leaves were washed and crushed prior to extraction. The air-dried leaves (5 kg) were extracted thrice with 30 L of 95% ethanol after maceration in 24 h. The supernatant was collected, combined and filtered through paper filter. Then the filtrate was concentrated in rotary evaporator to yield 238.6 g of a gummy residue, which was stored afterwards *in vacuo* in the dark.

Samples (1.0 g) of ethanol extract (EE) were reserved for pharmacological tests; the remaining crude EE was dissolved in 1500 mL water and extracted with petroleum, acetidin and *n*-butyl alcohol, successively, to afford 60.1 g (PE), 103.6 g (AE) and 45.5 g (*n*-butyl alcohol extraction, BE), respectively. The residue of extract was also been retained as the aqueous extraction (WE) for isolation. PE, AE and BE had been reserved 1.0 g for the following pharmacological screening.

PE (59.1 g) was subjected to silica gel column chromatography (12 × 70 cm), eluting with petroleum containing increasing amounts of acetidin with a flow rate of 16 mL/min to yield fraction A (petroleum:acetidin = 100:1, v/v), fraction B (100:3, v/v), fraction C (100:6, v/v). Fractions B and C were subject to silica gel, eluting with petroleum/acetidin gradient to obtain compound **4** (33 mg), **6** (21 mg) and **9** (18 mg) based on monitoring by thin layer chromatography (TLC).

AE (102.6 g) was subjected to silica gel column chromatography fraction using petroleum/acetidin/methanol as eluent to yield fractions D–F. Fraction D was further chromatographed eluting with petroleum/acetidin (100:20, v/v) to give sub-fraction D1, followed by separation on Agilent 1100 HPLC using a gradient of methanol/H_2_O/acetic acid (19:1:0.01, v/v) to give compound **1** (38 mg, *t*_R_ 12.9 min) and **2** (20 mg, *t*_R_ 20.3 min). Fraction E was further purified by preparative TLC and recrystallized to afford compound **3** (182 mg) and **5** (28 mg).

Parts of BE (44.5 g) were separated by MCI gel (5 × 40 cm, eluted with 30–70% MeOH) to obtain two fractions G and H, then compound **7** (32 mg, eluted with 40–60% MeOH) was purified from fraction G (68 mg), and compound **8** (20 mg, eluted with 40–60% MeOH) from fraction H (49 mg), respectively.

### Bioactive assay

PC12 cells were maintained at 37 °C in 5% CO_2_ in DMEM medium supplemented with 10% FBS, 150 U/mL penicillin and 50 μg/mL streptomycin before experiments. PC12 cells were seeded in 96-well culture plates (0.8 × 10^4^ cells per well) for 24 h. The resultant products were weighed, then resuspended with dimethyl sulphoxide (DMSO) at a predetermined concentration, so that DMSO content in culture medium, at concentration selected for the plant extracts, never exceeded 1% in the growth medium. At this concentration, DMSO had negligible effects on PC12. Cells were pretreated for 24 h with petroleum, acetidin, *n*-butyl alcohol and aqueous extracts of *P. decorate* at concentration of 0.1, 0.5, 1, 5 and 10 mg/mL. Then, 0.4 mM H_2_O_2_ was added in all samples for an additional 2 h except DMSO vehicle control. Cell viability was measured by the 3-(4,5-dimehthylthiazol-2-yl)-2,5-diphenyltetrazolium bromide (MTT) assay (Wong et al. 2012). Following treatment, the medium was removed and MTT solution (0.5 mg/mL in medium, Sigma Chemical Co., St. Louis, MO) was added. The incubation was kept for 4 h at 37 °C and then the culture medium was removed. Vitamin E (0.5 mg/mL) was used as positive control in MTT assay. Experiments were carried out in triplicate (*n* = 5). Absorbance was analysed at 490 nm with a Multiskan Ex microplate absorbance reader.

### Statistical analysis

Statistical analyses were done using one-way analysis of variance (ANOVA) with *t*-test. Values reported are mean ± SD of five repeats. Results were considered as statistical significance when *p* < 0.05.

## Results

### Structural elucidation

Compounds **1**-**9** were identified as betulin (**1**) (Wu et al. 2011), oleanolic acid (**2**) (Seebacher et al. 2003), ursolic acid (**3**) (Zhao et al. 2012), taraxerol (**4**) (Swasti et al. 2012), myricadiol (**5**) (Bi et al. 2015), 5,7-dimethoxy-4′-hydroxyflavone (**6**) (Zhen et al. 2000), β-daucosterol (**7**) (Hong et al. 2008), monotropein (**8**) (Bergeron et al. 1998) and lignoceric acid (**9**) (Li et al. 2006), by analysis of their NMR spectra and ESI-MS, and comparison with literature data. Betulin (**1**), myricadiol (**5**) and lignoceric acid (**9**) are reported from *P. decorate* leaves for the first time.

### Antioxidant activity against H_2_O_2_ induced cytotoxicity

The antioxidant capacities of *P. decorate* decoction, as well as the isolated phytochemicals, in protecting PC12 cells from hydrogen peroxide induced oxidative stress were investigated.

**Figure 2.**
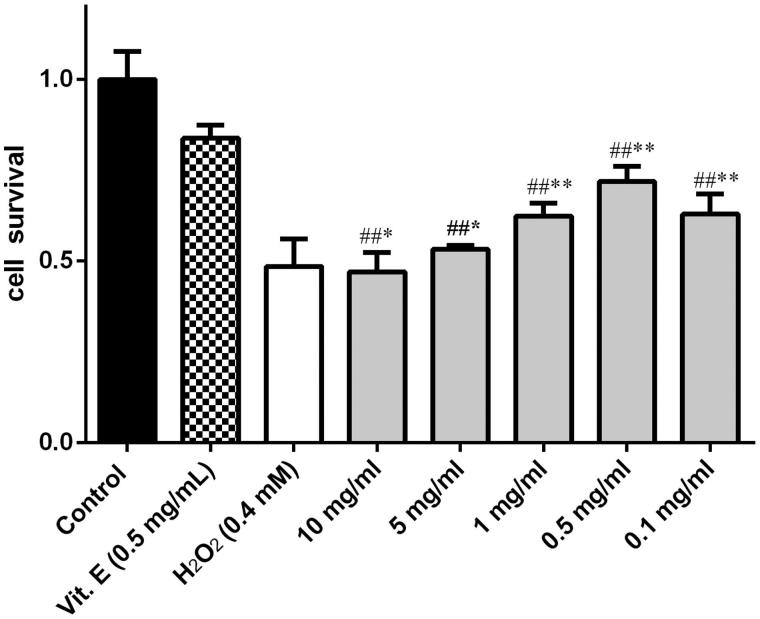
Protective effect of the alcohol extracts of *Pyrola decorate* on H_2_O_2_-induced cytotoxicity in cultured PC12 cells (means ± SD, *n* = 5). The data (cell viability, measured by MTT assay) were normalized and expressed as a percentage of the control group, which was set to 100%. Results were calculated from three independent experiments and are shown as mean ± SD. Compared with blank control group, #*p* < 0.05, ##*p* < 0.01; compared with H_2_O_2_ model group, *p < 0.05,**p < 0.01.

Figure 2 demonstrates 24 h treatment with 0.2 mM H_2_O_2_ alone produced significantly increased apoptosis (the H_2_O_2_ group 48%) compared with blank control group. Ethanol extract treated groups showed inhibitory activity with IC_50_ value of 10.83 mg/mL, inhibiting 72% of oxidative damage at 0.5 mg/mL concentration compared with 47% at 10 mg/mL. On the basis of these data, the 0.5 mg/mL of effective concentration of different polar extracts of *Pyrola decorate* were determined in the following experiments.

**Figure 3.**
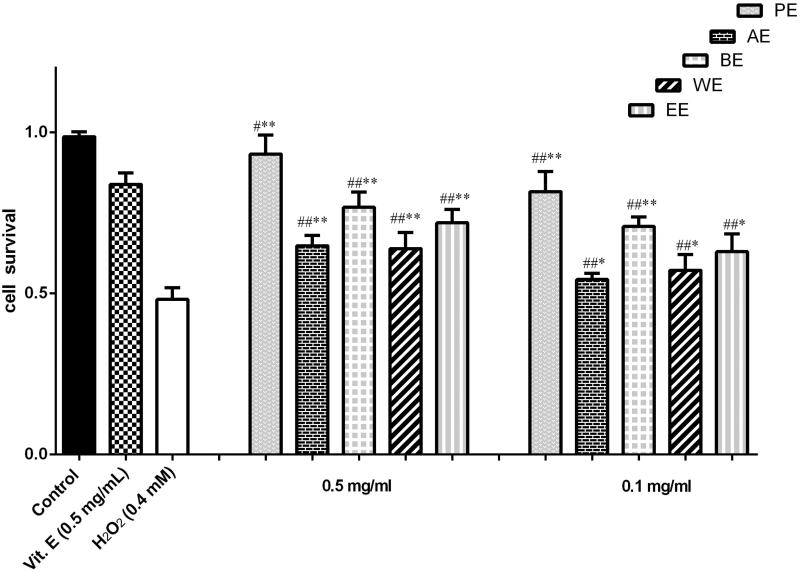
Protective effect of different polar extracts of *Pyrola decorate* on H_2_O_2_-induced cytotoxicity in cultured PC12 cells (means ± SD, *n* = 5). The data (cell viability, measured by MTT assay) were normalized and expressed as a percentage of the control group, which was set to 100%. Results were calculated from three independent experiments and are shown as mean ± SD. Compared with blank control group, #*p* < 0.05, ##*p* < 0.01; compared with H_2_O_2_ model group, *p < 0.05, **p < 0.01.

Figure 3 demonstrates the results that % cell viabilities were improved by different polar extracts of *Pyrola decorate* compared with the H_2_O_2_ treated cultures. PE and BE treated groups extracts showed better results when comparing with other extracts at the same concentration. PE (0.5 mg/mL) showed the strongest protective activities among all of the extractions (Figure 3), and yielded the maximum cell viability of 94% and 82% at the dose of 0.5 and 1.0 mg/mL, respectively. While BE extracts showed the higher cell viability of 77% at the dose of 0.5 mg/mL.

**Figure 4.**
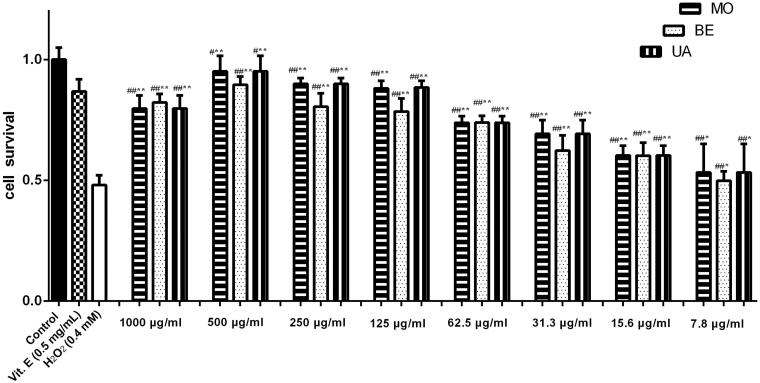
Protective effect of betulin (BE), ursolic acid (UA) and monotropein (MO), isolated from *Pyrola decorate*, on H_2_O_2_-induced cytotoxicity in cultured PC12 cells (means ± SD, *n* = 5). The data (cell viability, measured by MTT assay) were normalized and expressed as a percentage of the control group, which was set to 100%. Results were calculated from three independent experiments and are shown as mean ± SD. Compared with blank control group, #*p* < 0.05, ##*p* < 0.01; compared with H_2_O_2_ model group, *p < 0.05, **p < 0.01.

Three compounds promoted % cell viability from 7.8 to 1000 μg/mL concentration (Figure 4). All tested samples showed scavenging activity against H_2_O_2_ induced cytotoxicity of PC12 cells in a concentration-dependent manner. A significant potential for antioxidation of the tested phytochemicals was observed and three compounds had the stronger inhibitory effect compared with Vitamin E as a positive control agent. Betulin (**1**, BE), ursolic acid (**3**, UA) and monotropein (**8**, MO) were the main active constituents isolated from *P. decorate* leaves, and demonstrated with IC_50_ value of 6.88, 6.15 and 6.13 μg/mL, respectively.

The highest scavenging activity against H_2_O_2_ was observed in monotropein (95%, 500 μg/mL), depending on the concentration, the % cell viability of ursolic acid treated groups ranged from 53 to 95% within the concentration range of 7.8–500 μg/mL. As a result, ursolic acid, betulin and monotropein have also shown to enhance the protective effects against toxicity in PC12 cells. Simultaneously, three compounds isolated from *P. decorate* got their maximum efficacies at the concentration of 500 μg/mL.

## Discussion and conclusions

Oxidative stress causes endothelial dysfunction and cellular injury, which contribute to aging. Many studies indicate that oxidative stress from ROS has been widely implicated in aging related disorders (Saeidnia & Abdollahi 2013). H_2_O_2_ is a relatively stable reactive oxygen species (ROS) that is capable of diffusing through cellular membranes and induces production of O_2_·^–^ by activating NADPH oxidase (Ha et al. 2012). Treatment of PC12 cells with nontoxic concentrations of *P. decorate* extraction and the isolated phytochemicals could protect cells from H_2_O_2_-induced cytotoxicity with a decrease in the generation of ROS. In this study, EEs of *P. decorate* in different concentration had revealed the changes after treatment except in the maximum concentration of 10 μg/mL. The PE and BE extracts treated groups showed significant antioxidant capacities *in vitro* comparable with vitamin E as natural antioxidant, while the AE, WE and 50% EE extract groups exhibited relatively better antioxidant activity. The biological activities of the *P. decorate* extracts can be attributed to the secondary metabolites. 5,7-Dimethoxy-4′-hydroxyflavone was isolated from the PE extracts. Luteolin, kaempferol and 2′-*O*-galloyl-3-*β*-galactosyloxy quercetin were reported in the Pyrolaceae family by Zhen et al. (2016). Flavonoids, known as sensitive to oxidative stress, are higher in PE and lower both in BE and WE, were also found as the one of most commonly phytochemicals in *P. decorate* and exhibited obvious abilities of scavenging superoxide anion and hydroxyl free radical (Wang et al. 2014), and the effects of which came from their polyphenol structures (Sheng 2012). On the other hand, four of five triterpenoids in this study were isolated from the AE extract, it is noteworthy that individual compounds possessed remarkable antioxidant activities while the protection activity of AE extract against oxidative injury was not most significant, probably due to the interactions between heterogeneous ingredients even including that we have not achieved yet. BE extract also showed a better activity only second to petroleum ether compared with model group. Earlier studies have demonstrated iridoids were also one of the main biochemicals in *P. decorate* leaves (Liu et al. 2007), which are widely distributed among many medicinal plants possessing a range of biological activities (West et al. 2016). For example, evidence implied that the anti-aging effects of catalpol, an iridoid glycoside from *Rehmannia,* were achieved by promoting endogenous antioxidant enzyme activities and normalizing energy disturbance (Huang et al. 2016). However, the effect of iridoids in *P. decorate* targeting in the treatment of oxidative stress related neurological diseases has not been reported before.

As a continuous study, betulin, ursolic acid and monotropein isolated from *P. decorate* were investigated with capabilities of resistance to oxygen damage, which expanded our understanding of the antioxidant role of components candidates. Betulin was found from leaves of *P. decorate* for the first time. Ursolic acid was the most abundant and primary constituent of pentacyclic triterpenoid carboxylic acid in leaves of *P. decorate*, which has wide pharmacological effects of hepatoprotection, anti-inflammation, antitumor, antimicrobe and lipidemic regulation (Li et al. 2003). What is more, the neuroprotective therapeutic efficacy of ursolic acid has been confirmed by our previous studies (Yang et al. 2016). At cellular level, aging is associated with accumulating oxidative stress, mitochondrial dysfunction, telomere erosion and impaired DNA repair (Hung et al. 2010). One of the main mechanisms is the oxidation and antioxidation theory. The finding that ursolic acid played an important role to protect cells from oxidative injury suggested the consistency with our previous studies on its neuroprotection. Simultaneously, monotropein was one of the main components of the extracts from BE extract of *P. decorate* (Zhao & Tu 2010), and the bioactive tests indicated monotropein had possessed remarkable abilities against cytotoxicity induced by H_2_O_2_. In China, monotropein must be checked out in *P. decorate* as a crucial standard component in pharmacopeia. Furthermore, it is found *Pyrola* and *Morinda*, both are regarded as tonifying traditional medicines in China.

Oxidative stress and ROS are proposed to be major contributors to the aging process and many neurodegenerative diseases (Wang et al. 2016; Wojtunik-kulesza et al. 2016). Our study revealed that *P. decorate,* used as a tonic in traditional medicine, showed higher potency of anti-oxidative, which could partly explain the molecular mechanisms whereby *P. decorate* has neuroprotective effect of our previous study in neuronal cell models. In conclusion, *Pyrola decorate* is a potential antioxidative natural plant and worth testing for further pharmacological investigations in the treatment of oxidative stress related neurological disease.
